# The Property Rights Index (PRIF) can be used worldwide to compare different forest governance systems

**DOI:** 10.1038/s41598-023-46097-w

**Published:** 2023-11-09

**Authors:** Richard Rimoli, Liviu Nichiforel, Aditya Acharya, Alexandre Nollet, Bilal Snoussi, Lison Ambroise, Louis Cordonnier, Sandra Galván Mares, José Jonathan Aguirre Zúñiga, Jean-Daniel Bontemps, Laura Bouriaud

**Affiliations:** 1 Universitatea Stefan Cel Mare, Suceava, Romania; 2 Polcom Research and Consultancy Services, Kathmandu, Nepal; 3 AgroParisTech, Nancy, France; 4 Université Catholique de Louvain, Louvain-la-Neuve, Belgium; 5 Institute of Agricultural and Rural Sciences-Universidad Autónoma del Estado de México, Toluca, Mexico; 6 IGN–ENSG-Laboratoire d’inventaire Forestier, Nancy, France

**Keywords:** Ecosystem services, Environmental economics, Forestry

## Abstract

The bundle of forest landowners’ rights largely varies from one jurisdiction to another. On a global scale, the diversity of forest management regime and property rights systems is such that finding comprehensive and standardised approaches for governance analysis purposes is a challenging task. This paper explores the use of the Property Rights Index for Forestry (PRIF) as an analytical tool based on five rights domains (access, withdrawal, management, exclusion, and alienation) to assess how regulatory frameworks impact the owners’ forest property rights. We show that PRIF is a reliable index for various governance arrangements, considering its ability to score forest owners’ freedom to decide in case studies that range from the Amazon area (Brazil), Misiones province (Argentina) and Quebec (Canada) to community-managed Nepalese and Mexican forests. PRIF scores obtained in these diverse governance arrangements confirm that the governance of forests held by entities other than the state is driven by two factors: the owner’s ability to exclude the public from the use of his/her own resource and the owner’s freedom to decide on the forest management goals. These factors explained 66.44% of the variance in our sample and should be considered as the main potential drivers while implementing any new international or national policy. Despite having a few limitations, the PRIF is a promising governance indicator and has been proven to perform well for various socioeconomic and legal contexts.

## Introduction

Forests can be characterised by complex attributes representing assets for owners and other resource users. They produce a variety of goods and services, ranging from public to private goods in an economic sense^1–6,7^p. 2^8,9^.

In a broader system approach^10,11^, forest ownership is defined as a multi-layered system that interrelates distinct features, including the character of the owning entity (who is the legal owner of the resources), the nature of ownership that distinguishes between different property regimes, and the general institutional setting impacting the allocation of property rights (who can use the resource and under what conditions). Around the world, 22% of all forest area is privately owned by individuals, businesses, and communities, representing 887.7 million hectares worldwide. Meanwhile, public forests account for 73% of global forest area, 2% of which is managed by indigenous communities^12^.

The legal owners of the forest resources can be individuals, legal persons, communities, the state or other public/private institutions, corresponding to the main distinction usually made between private, common, and state property regimes respectively^13,14^. Private property is owned by a specific individual or private legal entity, while public property is owned, in the name of the citizens, either by the state (state property) or by local or regional administrative entities (e.g., municipal forests). Common property is owned conjointly by tenants. The common property regime is the institutional arrangement defining the conditions for the management and use of the resources that are used collectively^15,16^, pp. 15–16.

Being an owner implies having rights but also duties towards others. Rules and oversight are necessary to establish and enforce a property rights system^17^. Thus, characterising forest ownership entails the identification of the responsibilities (rights and duties) of actors that possess the sum of operational property rights (access and withdrawal) and collective choice rights (exclusion, management, and alienation). This characterization was first defined by Schlager and Ostrom^18^ and increasingly used in literature^19–25^. A reliable legal and institutional governance setup is a prerequisite for successful forest management, irrespective of the forestland ownership category^26–29^.

Property rights can be seen as social relations, defining the titleholder of something of value against all others^30–36^. To assess these rights is a challenging task as some of them are enforced by government (*de jure* rights) while others arise only from resource-use cooperation and are therefore not recognized by law (customary rights)^36–40^. Customary tenure arrangements are often informal and based on locally recognized rights without formal legal recognition. Still, as long as the legal entitlements are embedded in the constitutional setting of any country, the assessment of the rights based on a *de jure* situation provides an understanding of the role of the state in regulating the activities that can be performed by landowners.

The bundle of rights granted to landowners and their enforcement may vary between countries as a matter of social, historical, and economic settings^2^. This is reflected in different national and regional regulatory frameworks, defining what the forest owner and other resource users may or may not do in relation to forest goods and services^25^. Understanding these differences, from a cross-country comparative perspective, provides insights into the opportunities and challenges of implementing international and cross-sectoral policy objectives and commitments related to forest management.

Since 2005, The Rights and Resources Initiative (RRI) has been using an analytical framework to assess the impact of national laws that relate to the forest tenure rights of indigenous peoples and communities in Latin America, Asia and Africa^41^. At the core of the RRI resides the bundle of rights approach proposed by Schlager and Ostrom^18^. The RRI reports assess how far the communities can access forest resources (access rights), harvest timber or other forest products (withdrawal rights), make decisions regarding forest management (management rights), exclude outsiders from their forests (exclusion rights) and lease the resource, sell it or use it as collateral (alienation rights). However, the concept of RRI does not provide a frame for cross-country comparison and is limited to structuring the description of the diversity of institutional government-led arrangements to recognize the rights of Indigenous peoples and communities to forest resources. A specific index to depict the property rights situation globally has been developed by the Property Rights Alliance. Their Property Rights Index comparatively ranks countries on their legal and political environment, physical property rights, and intellectual property rights^42^, without distinguishing between the various types of natural resources, and being concerned only with private property rights.

In the case of Europe, the bundle of rights approach has been used in the case of individual private forest ownership. Nichiforel et al.^43^ have calculated an original property rights index in forestry (PRIF), designed to make a link between property rights distributions and the corresponding rule-of-law across different countries. In the initial study, PRIF values were calculated at the level of the legislation applicable in 31 European jurisdictions (countries and regions). The article pointed to important differences in the degree of freedom that forest owners have across Europe ranging from 84.7 degrees of freedom in the Netherlands to 38.4 in North Macedonia. In a subsequent paper, the PRIF index was used to identify the impact of the legal changes in property rights distribution in the last two decades, pointing out the stability of rights in most of the Central, Western and North European countries and the impact of the institutional changes in the Eastern European Countries^43^. The PRIF index has been used as an appropriate governance indicator in European reports and in large-scale assessments of the use of forest resources in Europe^44^. However, the applicability of the PRIF index to legal regimes outside of Europe was never tested. Besides, the PRIF is primarily designed for private forest owned by individuals, not considering other modalities of ownership, such as community forests. The extension to community forests could help to understand how governance works for this form of ownership that represents 28% of the developing world’s forests^45^ and is subject to increased vulnerabilities due to climate change and rapid societal transformation^46,47^.

In this study, we aim to test whether the categories and indicators analysed by the PRIF are universal enough to be adapted to any jurisdiction with different property rights arrangements and policy goals (production as well as protection). For this purpose, we tested the PRIF on a sample of five jurisdictions: two from South America (Argentina-Misiones, Brazil-Amazonas), two from North America (Canada-Quebec, Mexico) and one from Asia (Nepal), with a total forest cover of approximately 69.1 million ha^12,48–51^. Thus, the main research objective of the paper is to test the applicability of the PRIF in different institutional contexts, including different ownership forms and different regulatory frameworks. A subsequent research objective is to identify the impact of expected freedom limitations and particularities of each case study on the scores of the indicator and its categories. As an example of this hypothesis, variations are expected to be found in PRIF values linked to fewer management rights when analysing regulatory frameworks with stronger emphasis on forest protections, fewer alienation rights for community forests or greater exclusion rights for private forests. This study explores the methodological implications for further developing the PRIF as a universal analytical framework applicable across different forest institutional contexts and property regimes.

## Methods

### Five jurisdictions selected to exemplify how complex and diverse forest management regimes can be around the world

With the objective of testing the limits of the PRIF index, a broad mix of jurisdictions was selected so as to comply with at least two out of the four following criteria: (i) jurisdictions are not located in the European continent, (ii) jurisdiction are global forestry benchmarks for production and/or environmental importance, (iii) jurisdictions have forest management regimes with various policy or forestry objectives, (iv) jurisdictions are selected to represent diverse forests ownership categories.

All selected jurisdictions fulfil the first criterion. To fulfil the second criterion, Brazil and Canada were selected, as they consist of the second biggest forest area^12^ and the world’s largest forest product trade balance^52^, respectively. Canada also complies with our third point, as the country’s legislation allows for two types of forest management regime, as it will be explained further on. Also on this point, we selected the intermediary conservation classification in Misiones (Argentina), where there are three forest management classifications, according to the environmental protection status of the area. Lastly, for their diversity of forest ownership, we selected Nepal and Mexico, allowing us to test the capabilities of the PRIF index with community forests in two different continents and institutional contexts.

Argentina is a federal state divided into 23 provinces. Since the enactment of Law 26.331 in 2007, Argentina’s native forests have been divided into three categories, corresponding to different levels of conservation goals. Forest management is most flexible in low-priority conservation zones (green category), and wood extraction is forbidden in high-priority conservation zones (red category). Meanwhile, the intermediate level (yellow category), the one selected for analysis in this paper, allows for limited sustainable use, tourism, harvesting, and scientific research. The state analysed will be Misiones, which comprise 1.6 million hectares of native subtropical forest, 59% of which belongs to the yellow category^50^. Thus, this case study will serve as an example of the impact of forest protection legislation on the assessment of the PRIF indicators.

Brazil is a federal country formed by 26 states and a federal district. In this paper, the selected state was Amazonas, and the study only considers the management of “Reserva Legal” (RL). The RL is an area in which all rural landowners are obliged to have forested land, and its size varies according to the biome where the plot is located. In the Amazon Forest biome, landowners are obliged to have 80% of RL (permanent forest state), while the other 20% of the land can be changed at the owner’s discretion. The RL can be managed as long as land use does not change and follows Brazilian forest management laws. The exact amount of RL in the Amazonas state is not known. Using the data of the national rural land register (CAR) and knowing that all of the land situated in the state is in the Amazon biome, shows that there is a total of 68.2 million hectares of agricultural land in the state, meaning that there should be, roughly, 54.56 million hectares of RL in the studied state^49^.

Canada is a federal state composed of ten provinces. In Quebec, our selected province, the total amount of private forest represents 11.7 million hectares divided between 133,700 owners^48^. Forestry laws in this province distinguish between non-producers and producers, each having specific forestry regulations. “Producers” are owners who possess a forested area of at least 4 hectares who chose to follow a forest management plan certified by a forest engineer, allowing them to apply for forestry subsidies. This case represents 29,674 owners and 39% of the private forest in Quebec^53^. Meanwhile, “non-producers” are private forest owners that did not apply for the producer status, therefore, not being obliged to submit a forest management plan and, consequently, not having the possibility of applying for forestry subsidies. The study compares the legal distribution of rights for both categories of private forest owners (producers and non-producers) to identify the reliability of the PRIF methodology in capturing different institutional approaches of private forestry existing also at the sub-national level.

Mexico is a federal state in which the 1917 Constitution establishes three levels of government: the federal Union, state governments, and municipal governments. Currently, the General Law of Sustainable Forest Development, integrated into the Mexican Constitution regulates land management to achieve equitable development in the country and the promotion of agricultural, livestock and forestry activities. Article 27 sets guidelines for land tenure and property rights and establishes three property regimes: private property (individuals have control of their lands), public property (public entities with legal personality over assets in the public domain) and social property (composed of the *ejidos* and *comunidades*). Forests held by local, tribal and indigenous communities totalled 42.06 thousand hectares in 2015^51^. The *ejidos* have legal personality and fiscal obligations toward the State. The *comunidades* have the right to common lands for the economic sustenance of the entire communal land. Another form of common property defined by the Mexican constitution is the *ejido,* in which the members of a community are entitled to a portion of land to live on, a portion of land to cultivate and a portion of communal land. The internal rules of the *ejido* regulate the use, access, and conservation of the territory, as well as the rights and obligations of the *ejidatarios*. The general framework of property rights is similar between these two types of common property; thus, the study integrates the *ejidos* for the *de jure* assessment of the PRIF. The smaller differences with *comunidades* are presented in the results section.

Nepal is a federal state defined by its constitution as^54^ a country with a three-tier government structure; local, provincial, and federal. New laws, including forest laws, were adopted following the new constitution (2015) that modifies the decision-making powers of local and provincial government. However, the new forest law enacted in 2019 was not implemented through subsequent legislation at the time of the analysis. Thus, in the meantime, this analysis is based on the national and centralised laws that were currently prevailing in 2020. Nepal has two forest ownership types: private forest and national forest^55^. The community forests are part of the National Forests in which the management is done by local users, forming a User Group (UG), and has been an ongoing practice in Nepal since 1978^56^. According to the latest FRA report^12^, community forests accounted for 2237.67 thousand hectares in 2018. All the rights are given to the UG to manage the forest, but the land belongs to the state and its ownership cannot be changed by the group^57^. Article 67 of the Forest Act analyses how these rights are addressed in the law. In total, the forest area analysed by this study adds up to almost 70 million hectares (Table1).

**Table 1:** Characteristics of the selected jurisdictions.

Country	Argentina	Brazil	Canada	Mexico	Nepal
Jurisdiction	Misiones state	Amazonas state	Quebec	National	National
Category of property/conservation status	Private forest ownership	Private forest ownership	Private forest ownership	Community Forest	Community Forest
Forest management regime	yellow protection category	*“Reserva legal”*	Producers and non-producers	Use of forest by local communities	Use of forest by local communities
Total forested area encompassed by the study: ~ 69.51 million hectares	0.967 million ha^50^	~ 54.56 million hectares^49^	11.7 million hectares^48^	0,04 million hectares^51^	2.24 million hectares^12^

### PRIF methodology

The PRIF is based on 37 indicators (Appendix 1) that were formulated to cover the categories and subcategories of rights considering the practical possibilities that private forest owners have with their forest property, according to the law. The methodological foundation of the PRIF^43^ presents the steps used for data processing, data weighting and the aggregation of indicators in the calculation of the PRIF. The PRIF indicators are assessed based on formal legal rules (*de jure* applications of property rights). In its initial application, the indicators were assessed only for private forest ownership and for forests not located in protected areas, thus covering the usual practices regulated by the forest-specific legislation in a jurisdiction. “Jurisdiction” refers to a particular geographic area governed by a defined legal authority and can be a country if the forest legislation is unitarily applied at a national level or can specifically differentiate the areas where the forest legislation is set at subnational levels. Considering that each PRIF indicator contains a set of predefined alternatives based on the legal stipulations, we have checked the forest-specific legislation in the analysed non-European jurisdictions to see if the existing alternatives can also be found in these new jurisdictions. The list of alternatives was sorted by order of increasing restrictions on forest owners’ rights and were weighted from “no restrictions” (100 degrees of freedom) to “fully restricted” (0 degrees of freedom). Maximum or minimum degrees of freedom are theoretical possibilities for some indicators, but this method facilitates the weighting of the intermediate alternatives.

### Testing the PRIF on selected jurisdictions

The PRIF values were calculated for the legal regime applied to forest management in four federal states and one country from three different continents. Due to the structure of the legal system of each country, jurisdictions were analysed at a state or province level (Argentina-Misiones, Brazil-Amazonas, Canada-Quebec) or at a national level (Nepal and Mexico). When analysing the legislation of federal states, if no specific law was found regarding the matter under analysis, national state law was used instead, which happened with some frequency in Brazil, and sometimes in other cases. Only the legislation applicable to regular forest practices was considered, thus excluding special situations of forests from strictly protected areas or other restrictive zoning systems. For Argentina—Misiones, the legislation applicable for the yellow protection category was considered since this category provides for the economic utilisation of forest resources as opposed to strictly protected areas (red protection category).

The analysis of the legal texts (mainly the forestry laws of the studied countries and regions) aims to identify the legal provisions corresponding to each of the 37 PRIF indicators. To quantify the different levels of freedom in each jurisdiction, the authors used the multiple-choice assessment questionnaire^43^. It thus results in an evaluation of each indicator as addressed *de jure* in the forest regulatory framework. If there was no legislation for a specific indicator, the selected alternative was considered as “not regulated”, a situation that was also identified in the initial construction of the PRIF.

For the comparability of the interpretation of the legal text in the framework of the PRIF alternatives, a working group was set up with all country data providers, thus allowing for a common reiterative process in identifying the proper alternative for each indicator in each jurisdiction. The process took place in February 2020.

## Results

### Distribution of access rights

Only one indicator is integrated into the PRIF to analyse the distribution of access rights based on whether there is any restriction for the forest owners to enter their own forests (I1).

Argentina-Misiones and Brazil-Amazonas impose no restriction on the forest owners to enter their private forests; in the quantification of indicators, this means that the forest owners have the highest level of freedom regarding access rights assessed with 100 degrees of freedom. In Canada-Quebec, the ministry can impose restrictions under exceptional conditions, for example when lives are at risk because of forest fires. Freedom is scored at 90 degrees, as such restrictions are imposed only in exceptional circumstances and for the safety of the owners themselves.

In the case of the analysed common properties, access may be limited to different extents. In Mexico, access and transit are not restricted for the members of *ejidos* and *comunidades*, however, access can be limited if the rights are transferred to commercial entities by the decision of the communities. This situation falls under the indicators’ option with 80 degrees of freedom (“owners can negotiate the access restrictions and refuse them”). Nepal has the highest restrictions among the compared jurisdictions, as the officer in charge can prohibit the entrance of all individuals to the forest, including community forest members for a certain period^57^. This type of restriction is linked to the alternative “temporary imposed restrictions—owner cannot negotiate this” in the assessment of the indicator, which is considered as giving only 55 degrees of freedom of access to forest owners.

### Distribution of withdrawal rights

To assess the state involvement regarding the rights of an owner to harvest forest products and in what quantity, 11 indicators were used: six dealing with timber products and the other five with non-wood forest products (NWFPs).

Withdrawal rights for timber are largely influenced by the freedom of decision-making regarding the amount of timber to be harvested (I2). Thus, in four cases, this indicator provides no decision-making freedom to forest owners as the amount of timber is decided only by means of forest management plans (Argentina-Misiones, Quebec-producers, Nepal, and Mexico). In Nepal, a forest management plan is compulsory to harvest timber and to collect non-wood forest products, while in Mexico, forest technicians authorised by the governmental agency set the forest management plan that needs to be endorsed by said government agency. In Brazil-Amazonas, timber withdrawal is highly regulated, and owners’ rights are assessed with 15 degrees of freedom. In the case of Quebec, producers are obliged to follow a forest management plan (0 degree of freedom) while the non-producers can decide on the amount of timber to be harvested in the framework of general requirements, thus having the highest decision-making power of the analysed cases (90 degrees of freedom).

Regarding the approval of timber harvests (I3), restrictions to harvest apply in Nepal and approvals are needed for the other countries. In Nepal and Quebec, the owners can perform the harvesting by themselves (I6), in Brazil the owner is free up to a certain limit provided for in the law and, in Argentina-Misiones and Mexico, the owners need a licence to perform harvesting activities. This is reflected in the level of bureaucracy required to issue harvesting permits (I7) which is assessed as being a highly bureaucratic process in Argentina-Misiones and Brazil-Amazonas, rather bureaucratic for the community forests in Nepal and Mexico and a very easy procedure in Quebec, both for producers and non-producers.

**Figure 1:**
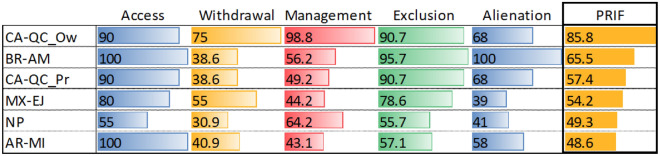
Overview of the Property Right Index in Forestry (PRIF): CA-QCow: Canada-Quebec non-producer; CA-QCpr: Canada-Quebec producer; BR-AM: Brazil-Amazonas; NP: Nepal; AR-MI: Argentina-Misiones; MX-EJ: Mexico-ejido, grouped into five property rights categories (PRCs). Each indicator was assessed on a scale ranging from 0 (“right fully restricted”) to 100 (“no restrictions apply”). The property rights index (PRIF, last column) is the mean of the 5 categories. The jurisdictions are oriented along the vertical axis and are sorted in decreasing order of PRIF values.

Considering the six indicators concerning withdrawal rights for timber, a large variation between the case studies was found in terms of the decision-making freedom of owners, non-producers in Quebec having the highest level of freedom (86.66) and user groups in Nepal the lowest (32.5) (Fig. 1).

Withdrawal rights for NWFPs present similar patterns to those for timber harvesting, with the highest value for the non-producer owners in Quebec (61) and the lowest in Nepal (30). Except for the non-producers in Quebec, results are quite homogeneous, with low scores for NWFPs (Fig. 1).

In Quebec, non-producers can harvest mushrooms for personal consumption (I8) and commercial use (I9) without any restrictions, but producers must act according to the forest management plan. In Argentina and Nepal, extracting NWFPs is allowed only in accordance with the management plan. In Brazil-Amazonas, general restrictions are provided for in the legislation, but no approval or planning are needed. In Mexico, all members of the communities and *ejidos* have the right to collect mushrooms, but the activity needs to be included in the approved forest management plan.

Game (I10) is considered to belong to nobody (*res nullius*) in Quebec, to the state of Brazil-Amazonas and Mexico, and to the hunting association in Argentina-Misiones. In Nepal, game is considered to belong *de jure* to the community forests, nevertheless, the members of the community cannot influence hunting activities. This situation is valid for all the case studies, hunting activities (I11) being strictly regulated, and owners having limited or no influence on it. In Mexico, the state, through technicians endorsed by SEMARNAT agency, establishes the hunting control rules. *Ejidos* and communities must respect endangered species, but hunting is allowed for subsistence and for animals used in traditional rituals. This *de jure* alternative identified for the community forests in the Mexican case study is a particular situation which was not considered in the initial designation of PRIF; nevertheless, the scoring for this category corresponds to the original PRIF alternative where “the owner can decide the amount of game to be hunted in the framework of an imposed maximum/minimum limit or state approval”.

Grazing (I12) is forbidden in Nepal, and no legal provisions were found in the text of laws of Argentina-Misiones and Quebec. For Brazil-Amazonas, general legal limitations apply while in Mexico the owners can make use of common lands for grazing, but the state can implement grazing rules in biodiversity rich forests.

### Distribution of management rights

Management rights have been evaluated with 13 indicators, divided into 3 sub-categories: rights for land use change, management planning, and implementation of forest management operations.

In the subcategory of land use change, both ownership categories from Canada-Quebec have total freedom, scoring 100 for the 3 indicators used in this subcategory. Meanwhile, Nepalese communities can only change forest land use when a portion of the land is donated to people in need, though the land still cannot be ploughed (I13). When the issue concerns the obligation of reforestation after clear cuttings (I14) and natural disasters (I15), Mexico has the lowest degree of freedom out of the six cases studied, not only having to reforest and maintain biodiversity after harvesting but also being obliged to restore and take conservation actions after natural disasters.

Management planning rights are low overall, Quebec-producers having the lowest score in the subcategory (23) considering their obligation to have a management plan, followed by Argentina (34). Only Nepal and Quebec Non-producers obtained over 50 degrees of freedom. Even then, Nepalese owners have limited rights regarding management planning. For example, it is only in Nepal that even the harvesting of brushwood needs the authorities’ approval in any circumstances (I16).

A full forestry management plan is needed for final felling (I17) in Mexico, Argentina, Brazil, and Quebec producers, the latter also not having any influence on the determination of goals in the FMP (I18). As for who is authorised to design the FMP (I19) Mexico has the lowest degree of freedom, SEMARNAT (State agency) accredited forest technicians being entitled to draw up the FMP, while in Nepal and Canada communities and, respectively, owners can do it by themselves. The approval of the document (I20) must be granted by the national forestry authority in Brazil, Nepal, and Argentina. Interestingly, Quebec producers have less freedom to maximise NWFP production (I21) when compared to the other countries.

Brazilian owners have more rights than Argentinian ones regarding operational management rights: their possibilities are even greater than those of Quebec producers (who can slightly influence rotation lengths and decide which species are to be used for reforestation within the framework of the law, neither of which Quebec producers can do). Nevertheless, Quebec non-producers are free to choose operational goals (I22), such as rotation length (I24), selection of trees to harvest (I23) and forest species to replant (I25).

Still, Nepal is more permissive than Brazil-Amazonas and Argentina-Misiones when it comes to operational management. Thus, Nepalese owners can decide rotation lengths (I24) based merely on technical provisions, as well as manage the forest by themselves without the help of professional consulting. Users are not allowed to sell the timber for commercial purposes outside the group before fulfilling the demand within the group, as will be seen further along in alienation rights. In Nepal, in general, market demand for timber does not influence forest management.

This is also true for operational management: Quebec producers need a forest management plan to determine the amount of wood to cut if they want to receive government subsidies. Non-producers, on the other hand, are completely exempt from having such a document. This contrasts with the situation of Brazil-Amazonas, Argentina-Misiones, and Nepal in which a forest management plan is mandatory, regardless of the forestry work, and the cost of drawing up the FMP is to be paid by the owner.

In sum, concerning the overall management rights, rights are highly restricted in Argentina-Misiones, followed by Mexican *ejidos* and producers in Quebec. Then comes Brazil-Amazonas (moderate restrictions) and Nepal (a little more permissive). Non-producers in Quebec have almost complete freedom. For example, both categories in Quebec were the only ones in our study that have no reforestation obligation, neither after final cutting nor after natural catastrophes (Fig. 1).

### Distribution of exclusion rights

Exclusion rights refers to the owners’ legal abilities to prevent external users from entering the property and benefiting from the forest resources. To measure these rights, seven indicators were used assessing the legal rights of owners to exclude: access to the forest, hunting, camping in the forests, forest road use restrictions and prohibition for users to harvest mushrooms or other NWFPs, for recreational or for commercial use.

In the subcategory of public access, Brazil-Amazonas, Canada-Quebec (both categories) and Mexico have the highest score when it comes to the restriction of access to the property for recreational purpose (I26) (100, 100 and 90 respectively). No regulations were found for Nepal and Argentina on this topic, therefore both got only 30 degrees of freedom as in the original setting of the PRIF. Legislation on the restriction of access to forest roads crossing the property (I27) was only found in Brazil-Amazonas and México (70 and 75, in that order). Camping (I28) can be fully restricted by forest owners in Brazil and both Quebec categories (100), while this indicator is not regulated in Argentina (50) and only partial restrictions can be applied in Nepal and Mexico community forests.

The second subcategory of exclusion rights concerns the exclusion of public use of NWFPs. Brazil-Amazonas and Canada-Quebec offer full rights to owners, averaging 100 in this subcategory with four indicators. Recreational mushroom picking (I29) in Argentina is considered “everyman’s right”, therefore, forest owners cannot prevent members of the public from doing it. Meanwhile, in Nepal and Mexico, the communities have the possibility of putting up signs letting outsiders know that there are some restrictions, corresponding to 80 degrees of freedom. All countries but Mexico have full freedom to restrict commercial picking (I30). Mexico scores the same (80 degrees of freedom) for commercial and recreational picking. Hunting (I31) can be fully restricted by forest owners in Brazil-Amazonas, Argentina-Misiones and both instances in Canada-Quebec. In Mexico the *Ejidos* can define an area where hunting is allowed with some restrictions (80 degrees of freedom), while in Nepal hunting is forbidden in national forests, therefore 0 degrees of freedom was attributed to this indicator. Lastly, fencing of the forest (I32) is allowed by law in every country (100 degrees of freedom), except in Nepal, where no legislation was found on the matter.

Thus, Brazil-Amazonas (95.7 degrees of freedom), followed by Canada-Quebec, producers and non-producers (90.7 degrees of freedom), have the highest exclusion rights, having almost complete freedom to exclude. On the other hand, in Nepal (55.7 degrees of freedom) and Argentina-Misiones (55.7 degrees of freedom) owners have relatively low rights to exclude (Fig. 1). In the methodological setting of the PRIF, “exclusion rights” is the only category where no regulation does not mean absolute freedom, as it brings a certain legal uncertainty to the landowner due to his/her rights for exclusion not being clearly set out. This is especially true in Misiones-Argentina, where four out of the seven indicators were not regulated. Comparable results were found in Nepal (two out of seven indicators of exclusion rights are not regulated).

### Distribution of alienation rights

In Nepal and Mexico, alienation rights (I33) belong to the state as a specific legal setting regulating community forests. On the other hand, full alienation rights are permissible in Brazil-Amazonas. It is the only jurisdiction, among those studied, where owners can sell forest land without any restrictions. In Canada-Quebec, it is required that the state be informed before selling one's forest and permission for the sale can be denied. As for Argentina-Misiones, there is no need to inform the state but there are also some restrictions to selling. There is a minimum and maximum price for forest land (I34) in Argentina-Misiones depending on specific conditions, at the discretion of the provincial court.

Only owners from the case studies from Brazil and Mexico have total freedom to decide to whom to sell timber (I35), while the remaining owners have restrictions. All the jurisdictions studied, except for Nepal, allow owners to choose the form of selling timber (I36), but the price (I37) is imposed for some markets in the case studies from Mexico and Canada-Quebec, and always imposed in Argentina-Misiones.

The average degree of freedom for alienation rights for the six analysed jurisdictions is 69 (Fig. 1).

### Property rights index in forestry (PRIF)

The PRIF and the five categories of property rights allow the comparison of the distribution of property rights across the different jurisdictions. This study shows clear variations in decision-making for forest owners across the analysed jurisdictions. The PRIF ranges from 48.6 degrees of freedom in Argentina-Misiones to 85.8 degrees of freedom in Canada-Quebec non-producers case study (Fig. 1). The combination of withdrawal, management and exclusion explains the diversity in PRIF scores across those countries and highlights differences in the policy process.

The overall assessment of the PRIF points to the differences in the assumed selection of the case studies. The lowest overall score in the Argentina-Misiones case study is linked to the reduced scores for withdrawal and management rights considering the greater emphasis on forest protection corresponding to the intermediate level yellow forest protection category selected for the analysis. On the other hand, the non-producer owners from Canada-Quebec have the highest scores for withdrawal and management thus showing the amount of freedom given in this jurisdiction to those owners that do not want to apply for state subsidies.

It can be observed that owners from Canada-Quebec and Brazil-Amazonas have more freedom to exclude people and change the land use than the owners from other jurisdictions. Forest owners have, for example, almost full exclusion rights in Canada-Quebec and Brazil-Amazonas while these same rights are very restricted in Nepal and Argentina-Misiones.

When looking at the case studies selected from Canada-Quebec, the PRIF explains well the differences between the non-producer owners, that have greater management and withdrawal rights compared to the producers.

### Factors detailing the scores identified with Principal Component Analysis (PCA)

**Figure 2:**
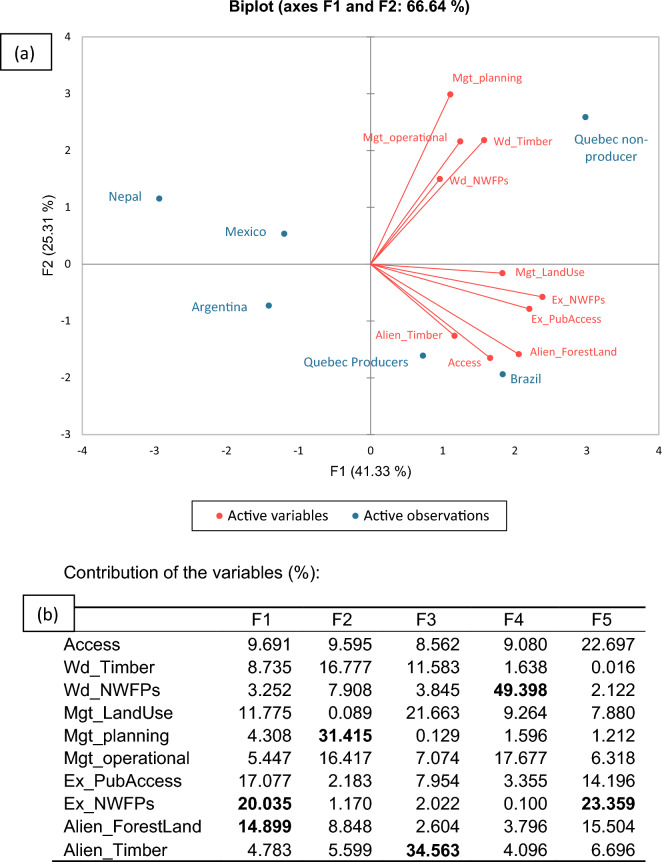
Principal component analysis of the property rights distribution in forestry. The analysis considers the values identified for the 10 sub-categories of rights as variable: Access rights (Access), withdrawal rights for timber (Wd_Timber), withdrawal rights for non-wood forest products (Wd_NWFPs), rights of land use change (Mgt_Land use), rights of management planning (Mgt_Planning), rights of operational management (Mgt_Operational), exclusion of public access (Ex_PubAccess), exclusion for the use of NWFPs (Ex_NWFPs), alienation rights for forest land (Alien_Forestland) and alienation rights for timber (Alien_Timber). Part (**a**) refers to the correlation circle and the individual factor map and part (**b**) depicts the variables’ contribution. The first factor (F1) is freedom to restrict public access and to restrict withdrawal of NWFPs, and the second factor (F2) is freedom to formulate management goals and to make decisions regarding operational management.

To identify the role of each subcategory of rights in differentiating the selected case studies, a Principal component analysis (PCA) was performed on the ten variables describing PRIF (Fig. 2).

The analysis was carried out regarding the 37 indicators grouped into 10 sub-categories of rights. The six case-studies from the five jurisdictions were projected on the first two principal components that, together, explain 66.64% of the variance (Fig. 2a).

The first principal component (F1, representing 41.33% of total variance) is explained by indicators on exclusion for the use of NWFP (20.0%), exclusion of public access (17.1%) and alienation rights for forest land (14.9%) (Fig. 2b). The freedom to make decisions on those exclusion rights explains most of the variation of the PRIF regarding jurisdictions and is the main source of variation between countries (Fig. 2b). Thus, the first factor is explained by the owners’ freedom to restrict withdrawal of NWFP and to restrict public access, tending to increase from left to right.

The second principal component (F2, representing 25,31% of the total variance) is highly determined by management rights (management planning explains 31.4% of total variance while operational management, 16.4%) but also by withdrawal rights for timber (16.8%). The second factor depicts the owners’ freedom to formulate management goals and to make operational management decisions. These categories differentiate between the jurisdictions in the upper half, that have fewer alienation but more management rights, and those in the lower half.

The analysis of each quadrant and their diagonals, in this case, permits a better understanding of the PCA characteristics and components. The first quadrant (upper-right) is characterized by a high degree of management and withdrawal freedom, exemplified by Quebec non-producers, who are not obliged to follow a FMP. Argentina-Misiones is on the opposite side of this diagonal (quadrant 3). In this jurisdiction, the forests analysed are partially protected (yellow category) and have high conservation values, the legal regime therefore being the most restrictive when it comes to management and withdrawal rights. This diagonal explains the different legal approaches impacting on owners’ rights when the forestry goals are production-oriented versus conservation-oriented.

Meanwhile, the jurisdiction distribution along the second diagonal is explained by alienation, exclusion, and access rights. Jurisdictions from the second quadrant (lower right) are those with higher scores in these rights, while at the opposite end we can find jurisdictions with lower scores (community forests from Nepal and Mexico). This diagonal can be framed as community versus individual rights in forest utilisation.

## Discussion

While the PRIF has been methodologically designed for the specific legal situation of European private owners, this study shows that the PRIF can be applied to countries outside Europe, despite their different legal and governance arrangements. The study confirms that the PRIF methodology can be used outside Europe to perform worldwide comparative analyses. First, it was confirmed that the questionnaire was applicable for each indicator composing PRIF in all the jurisdictions. This points to the fact that the list of indicators selected to differentiate between the five rights categories is universal enough to be implemented across different property regimes in different regulatory contexts. Secondly, the list of alternatives provided for each indicator was found to be sufficiently detailed to cover the variations between countries and to score each indicator. Nevertheless, the terminology of the alternatives may need to be adapted to the local property rights regime. One example was the right of Mexican *ejidos* to hunt animals for subsistence or rituals, an alternative which is not found in the original design of the PRIF, but which was covered in a satisfactory manner by the existing alternative “the owner can decide the amount of game to be hunted in the framework of an imposed maximum/minimum limit or state approval”.

In order to contextualise the results of the analysis, a comparison with the results published by Nichiforel et. al.^43^ is provided for the PRIF values (Appendix 2) and for the PCA analysis (Appendix 3) with the caveat that the European PRIF values were analysed at the 2015 level while our study was performed considering the legislation applicable in 2020. However, legal norms are relatively stable institutions as argued by^58^. Only 2 out of the 28 (7%) analysed legal texts were modified after 2015.

The comparison with Nichiforel et al*.*^43^ points to three arguments validating the PRIF methodology: (i) The six PRIF scores of the added jurisdictions are well spread among the values of the European countries (Appendix 2). (ii) The variables factor map (Appendix 3) is well integrated amongst other scores (iii) No jurisdiction appears as an outlier, though their particularities where clearly reflected in their scores of PRIF categories. Moreover, the main factors resulting from the PCA analysis differentiating the jurisdictions are similar in the European cross-jurisdiction comparison^43^ with those identified in our analysis (Fig. 2), albeit in reverse order. Thus, the freedom to make decisions in operational management and to formulate management goals appears as the first differentiating factor in the European-based sample of jurisdictions, for which it has a stronger explanation value than in our case.

Comparing the PRIF scores, the Quebec-non producers’ case study is rated at 98.8 degrees of freedom, this PRIF value being the highest when putting together all jurisdictions (from this study and from the European sample^43^) while the owners from the Argentinian case study still enjoy a slightly higher level of freedom that the private owners from some Eastern-European countries (e.g., Bulgaria, Romania). Having a closer look at the land use management category, it appears that, within the added jurisdictions, unlike many European countries, there is no timeframe set for the owner’s reforestation obligation after final cutting (Nepal, Argentina-Misiones, Brazil-Amazonas), or even no reforestation obligation at all (Canada-Quebec producers and non-producers). This is still true in the case of reforestation after natural catastrophes (I15), except in Brazil-Amazonas, where the owner is exempted from the reforestation obligation. As a result, all these added jurisdictions (except for Nepal and Mexico, due to the nature of community forests) have a higher score in land use management, which reflects the high degree of liberty of the forest owners concerned. It seems that this variable differentiates to the highest degree the non-European jurisdictions from the European ones.

Land use management is quite distinct from the other kinds of management. Galik and Jagger^59^ suggest adding to Ostrom and Schlager’s Framework a 6th right category, named “Alteration”, that corresponds to “the right to change the set of goods and services provided by a resource”. In our opinion, this right is an intermediate category between the management right and the alienation right. If the alienation right is understood to be the landowner’s ability to change the very nature of the good owned (the good’s essential attribute), the ‘alteration’ can depict more nuanced situations in which the nature of the good remains the same, e.g., forests, but the landowner can decide on the amount and type of services his/her forest provides. This is a critical distinction at the heart of the European debate on nature restoration law^60^, e.g., to what extent the forest owner has the right to change essential characteristics of the forests such naturality, diversity or connectivity without these changes being considered forest deterioration. In this respect, PRIF-based analyses could bring relevant qualitative information about the existing set of rights in forest management that will influence the implementation of any new international or national policy.

Up to now, the PRIF had been applied only to individual forest ownership. The studied framework has now been successfully applied also to community forests within Nepal and Mexico. This paves the way for a comparison between different community forest jurisdictions but also between other types of forest management jurisdictions, such as Joint Forest Management^61^. According to^62^, *de jure* rights are among the five most frequent variables explaining social and environmental outcomes. Therefore, it is important to take them into account, whether it is about understanding and assessing community forests’ management benefits or their disadvantages. To compute the PRIF indicator of these forests in Nepal and Mexico, the users were considered as proprietors, as per^63^. In many situations, not all the users contribute equally to decision-making^64^. This phenomenon has already been observed in community forest literature^65,66^. For example, while Nepal and Mexican *Ejidos* have similar PRIF values (49.3 and 54.2, respectively), the composition of the 5 categories was quite different. As expected, alienation rights were low due to the nature of community ownership. For access rights, Nepal has more instances where the state can restrict the community members’ access to their forest land. In the meantime, management rights are greater in Nepal, especially in the subcategory of management operations, where Nepal scores 64.2 and Mexico 44.2. Thus, the PRIF allows a fine qualitative analysis of legal decision-making power for similar forms of governance.

As shown in Fig. 2a, the second dimension of the PCA is mainly related to forest management, which means this is one aspect that strongly differentiates the jurisdictions. In the case of Quebec, the distinct rules for “non-producers” and “producers” are particularly interesting and well captured by Fig. 2a. “Non-producer” owners are relatively free to manage their forests, whereas producers must follow a forest management plan (FMP) to obtain funding. FMPs are important tools for forest agencies to influence forest landowners’ behaviour and implement forest policies. They can be seen as a policy instrument, i.e., a structured effort by governors to modify the actions of the governed, and can be classified as “sticks, carrots or sermons”, depending on how they are implemented^67^. In Nepal, Argentina-Misiones and Brazil-Amazonas, FMPs mainly operate as a “stick”, since they are mandatory to exploit goods from the property, in certain agreed modalities, and sanctions may apply if not observed. In this sense, they fulfil a regulatory function, within a paternalistic state paradigm^68^ and rationalist planning approach^69^. In this situation, state regulation curbs the owners’ ability to use their knowledge to manage the forest^2^, which is reflected in lower values of the PRIF, but make room for other stakeholders’ claims upon forest goods and services^70^.

In Quebec, forest owners that chose to be “producers” are required to have FMPs to benefit from economic incentives, and thus the FMP plays the role of a “carrot”. However, as soon as an owner decides to adhere to an FMP, some management rights are transferred *de jure* to the forest agency, which may influence forest owners’ motivation to apply. Consequentially these subsidies can have limited success if informational and educational actions are neglected by government agencies^67,71^. Thus, PRIF may contribute to a deeper understanding of private forest owners' motivation to adhere to different payment schemes, while they affect their overall bundle of rights in forest management.

Through the division of property rights into five property right categories, the PRIF can enable researchers to better understand how the rule-of-law interferes with the substantive content of the ownership. Secondly, PRIF indicators allow a comprehensive analysis at different levels of decision-making, from a constitutional level (which is often the case of access and exclusion rights), thus deeply rooted in public policies to an operational level (which is the case of harvesting quantity and mushroom picking rules). Additionally, the PRIF provides a structured overview of property rights differences between countries. It can transform various legal norms and rules into one integrative index and open the way to a deeper institutional analysis. Besides, the temporal dynamic of the index is easy to access as long as the traceability of legal changes subsists. Therefore, the establishment of a common PRIF index for other countries, as seen above, should be possible and advisable. The PRIF can mark a big step forward, as the index is able to depict a particular regime in forested area with a homogenous institutional environment, e.g., form of ownership, timber production goal, conservation regime, etc., as opposed to the current practice of focusing solely on one jurisdiction-related legislation. For example, countries such Brazil, Argentina, Mexico, and Canada have different rights in each federal state/province. Moreover, for the same state/province, as is the situation in Canada – Quebec, the regime applying to a certain forest area may be completely different as shown in the case of producer/non-producer forest owners. Thus, a very accurate analysis of each state/province and of each forest regime in place for different forms of private ownership can be done.

A few constraints can be pointed out concerning the PRIF. Firstly, the PRIF only works on *de jure* governance and can conflict with actual practices, especially in countries affected by corruption or where informal governance mechanisms (e.g., customary rights, illegal forest extraction) are predominant^72^. Many private areas in Brazil, for example, but also in Europe, witness failures to comply with the law, and alternative approaches to regulation are under discussion, such as the pay-for performance approach^73,74^ or result-based payments^75^. Indeed, huge discrepancies may exist somewhere between the rule of law and the actual implementation of the law, an issue highlighted in literature as law or policy enforcement failure^76^, policy implementation gap^77–79^ *de jure* and de facto rights^10,80–83^ or as institutional voids^84^.However, our objective was to understand if the PRIF can be used to disentangle the legal regimes applying to private forests outside Europe and to community forests. At this stage, the PRIF is scoring the ‘*de jure*’ situation and shows that the content of the property rights as “legal entitlement by law” or “right to act” may vary from one country to another or even within a country. The next step will be to distinguish^85^ between the economic property rights and legal property rights, e.g., what an owner can effectively do versus what an owner is entitled to do. In fact, analysing the tension between ‘*de jure*’ and ‘de facto’ property rights is a promising way of researching institutional entrepreneurship, or for improving policy implementation^86–88^. Therefore, the PRIF could be adapted to assess the gap between legal property rights and economic property rights, if it is thought to effectively score the level of operational freedom that owners have.

Secondly, the PRIF does not consider market-driven governance mechanisms^89^. This is the case when FMPs are not compulsory, but the owner decides to adhere to forest certification schemes, leading him to follow market-driven forest standards. However, the impact of the market-driven governance mechanisms on the property rights can be assessed, as in the case of the voluntary FMPs (Canada-Quebec producers’ category). Furthermore, unclear laws can pave the way for different interpretations, and some indicators might be less relevant depending on their context such as grazing and tree. For example, in Quebec, “tree silviculture” is only applied for experimental purposes and grazing under tree cover is less common than in Europe, thus not regulated.

Finally, a constraint to be acknowledged is that the PRIF has high reliability and provides good explanatory values when it is calculated for forest areas with similar management goals, e.g., primary economic functions or nature conservation functions allowing a certain level of management actions that can be decided by the owner. This precaution will reduce the potential bias that contrasting management goals may generate and will provide more reliability to data comparison.

This paper proves the possibility of extending the applicability of the PRIF outside Europe and to distinct types of ownership, particularly to community forests. The latter represent 14% of the total forest area in the world and are the object of a constantly renewed interest for researchers. Whether it is about assessing the best system for forest conservation or exploring the benefits and drawbacks of community forest management on the living conditions of the community members, community forests are a growing field of interest. The fact that the PRIF was successfully applied to Nepal and Mexico community forests paves the way for future research to apply this tool to other community forest jurisdictions, but, even though the list of indicators used was applicable to the cases analysed in this paper, the global diversity of the internal rules for community forest management might require modifications and the addition of more specific indicators to the current PRIF methodology.

The preliminary studies done to test the PRIF adaptation to the context of protected area management in Europe show the potential of the PRIF to identify the level of restriction on property rights between different types of conservation zones, also applied to^90^. This broadens the applicability of the PRIF to research conducted in different directions. One way is to compare the level of restrictions on property rights in the same jurisdictions considering different conservation requirements such as, for example, for the three zones existing in Argentina. This can provide stronger arguments to obtain for financial support for forests with conservation goals to compensate the owners for the level of restrictions imposed, in contrast with the absence of restrictions imposed on owners of forests with economic goals. Another applicability is to use cross-country comparisons between legal requirements imposed for areas with the same conservation status (e.g., to compare restrictions imposed in Natura2000 protected areas across different European states). The results show that PRIF is a powerful analytical tool that enables the cross-country comparison between different forest legal regimes. As a way of further exploring the PRIF potential, future research could also look at how the index would fare being used as an environmental/management governance indicator and how it compares to other well-established governance indicators.

### Supplementary Information

Supplementary Information 1.

Supplementary Information 2.

## Data Availability

All data generated or analysed during this study are included in this published article [and its supplementary information files].

## Appendices

### Appendix 1

**Table:**

Code	Sub-categories	Categories	Issue assessed
I1	Access	Access	Restrictions on owners to enter their own property
I2	Timber	Withdrawal	Scope of decision on the amount of wood to be harvested
I3			Approvals that owners need to harvest timber
I4			Scope of decision for brushwood
I5			Approvals that owners need to harvest brushwood
I6			Legal possibility to perform the timber harvesting operation
I7			Rigour of bureaucratic procedures to get harvesting permits for timber removal
I8	NWFP		Restriction on owners to harvest mushrooms for their personal consumption
I9			Restriction on owners to harvest mushrooms for commercial use
I10			Ownership on game/wild animals in a private forest
I11			Scope of decisions on the amount of game that can be hunted from a private forest
I12			How are the rights of grazing in the private forest regulated?
I13	Land use change	Management	Scope of decision to change the forest land use
I14			Obligation for reforestation of forest lands after final cutting
I15			Obligation for reforestation of forest lands after natural catastrophes
I16	Management planning		Request for a forest management planning (FMP) for private forests
I17			Types of planning documents required for the final felling
I18			Integration of owner’s goals into the FMP
I19			Authorized persons to design the FMP for private forests
I20			Approval of the FMP for private forests of individuals
I21	Implementation of forest management operations		Scope of decision for abandoning the timber production and producing NWFPs
I22			Technical expertise for the implementation of forest operations
I23			Scope of decision on the selection of trees to be harvested
I24			Scope of decision on the rotation length
I25			Scope of decision on the type of species to be used for reforestation
I26	Public access	Exclusion	Scope of decision to restrict the public access for recreational purposes
I27			Scope of decision to restrict the access on forest road crossing the property
I28			Scope of decision to exclude non-owners from camping in the forest
I29	Use of NWFP		Scope of decision to exclude the public from the recreational harvesting of mushrooms
I30			Scope of decision to exclude others from the commercial harvesting of mushrooms
I31			Scope of decision on how hunting activity take place in a private forest
I32			Legal requirements in respect to fencing the private forests
I33	Land	Alienation	Scope of decision in setting the forestland
I34			Scope of decision in setting the price of forestland
I35	Timber		Scope of decision for selling the timber
I36			Scope of decision on the form of timber commercialisation
I37			Scope of decision on the price to sell the timber

### Appendix 2

Overview of the Property Right Index in Forestry (PRIF), with data from^43^ and our selected non-European jurisdictions: CA-QCow: Canada-Quebec non-producer; CA-QCpr: Canada-Quebec producer; BR-AM: Brazil-Amazonas; NP: Nepal; AR-MI: Argentina-Misiones; MX-EJ: Mexico-ejido, grouped into five property rights categories (PRCs). Each indicator was assessed on a scale ranging from 0 (“right fully restricted”) to 100 (“no restrictions apply”). The property rights index (PRIF, last column) is the mean of the 37 indicators. The jurisdictions are oriented along the vertical axis and are sorted in order of decreasing PRIF from the top down.

### Appendix 3

PCA joining data from this paper and Nichiforel et. al.^43^
